# Laparoscopic Transabdominal Preperitoneal Repair of a Primary Upper Lumbar Hernia: A Case Report

**DOI:** 10.7759/cureus.79007

**Published:** 2025-02-14

**Authors:** Ioannis Katsarelas, Dimitrios Chatzinas, Ismini Kountouri, Dimitra Manolakaki, Miltiadis Chandolias

**Affiliations:** 1 Department of Surgery, General Hospital of Katerini, Katerini, GRC

**Keywords:** general and laparoscopic surgery, grynfeltt hernia, grynfeltt-lesshaft hernia, primary lumbar hernia, transabdominal preperitoneal (tapp)

## Abstract

Lumbar hernias are a rare type of hernia, arising through posterolateral abdominal wall defects containing either intraperitoneal or extraperitoneal contents. Most lumbar hernias are primary, incisional, or trauma-related, while congenital lumbar hernias are uncommon. Surgical management comes down to two approaches: the classic repair technique with an open approach utilizing a lumbar incision and the laparoscopic approach, either transabdominal preperitoneal (TAPP) or totally extraperitoneal (TEP). We present the case of a 56-year-old female patient who was evaluated in the outpatient surgical clinic, complaining of pain located between the left midaxillary line and the left lumbar region. The patient underwent a CT scan, and a diagnosis of a left upper lumbar hernia was made. The patient underwent a laparoscopic TAPP repair and was discharged on postoperative day one. Our case highlights that lumbar hernias can present with vague symptoms and without an obvious lump/mass, a diagnosis of which could require a great degree of clinical suspicion especially when there is no history of trauma or surgery in the area. Early imaging can confirm the diagnosis, and surgical repair should be conducted to prevent possible complications.

## Introduction

Lumbar hernia is a rare type of hernia accounting for less than 1.5% of primary abdominal hernias [1]. Defects are located in two triangles located in the lumbar region. The Grynfeltt-Lesshaft triangle (superior lumbar triangle) is an inverted triangle whose borders are the 12th rib superiorly, the posterior border of the internal oblique muscle laterally, and the quadratus lumborum muscle medially. Superficially, this region is covered by the great dorsi muscle, and its floor is formed by the aponeurosis of the transverse abdominis muscle. Petit's triangle (inferior lumbar triangle) is an upright triangle bordered by the iliac crest inferiorly, the external oblique muscle anteriorly, and the latissimus dorsi muscle posteriorly. The floor of Petit's triangle is formed by the aponeurosis of the internal oblique and transversus abdominis muscle and it is superficially covered by subcutaneous fat and skin [2]. Generally, superior lumbar hernias are more common than inferior lumbar hernias. The complex anatomy of the lumbar region, including its muscular and fascial layers, contributes to the unique characteristics of lumbar hernias. Such hernias are either acquired in 80% of the cases (trauma, incisional, primary) or congenital in 20% of the cases. Lumbar hernia is frequently asymptomatic but may be associated with nausea, vomiting, renal deficiency, pain, and swelling in the lumbar region [3]. Diagnosis of a lumbar hernia is primarily clinical through palpation of the lumbar area with evidence of local swelling or by volume increase while utilizing a Valsalva maneuver; however, many cases can be challenging especially in overweight or obese patients [4]. In cases where no mass or defect can be palpated, a CT scan is the gold standard for the diagnosis and evaluation of the herniated contents [5]. Differential diagnosis must be made by other similar clinical entities such as lipomas, fibromas, hematomas, abscesses, kidney tumors, muscle hernia, and panniculitis [6]. Surgical repair is the only curative treatment either by a lumbarotomy (open approach) or laparoscopically. In both of the abovementioned techniques, the surgeons' aim is to free the muscle wall defect by reducing the contents of the hernia, whether it is an organ or adipose tissue, and to proceed with the repair of the muscle wall primarily by using a synthetic mesh. Every case of lumbar hernia has unique characteristics, and it is up to the surgical team to proceed with the optimal approach of repair.

## Case presentation

**Figure 1:**
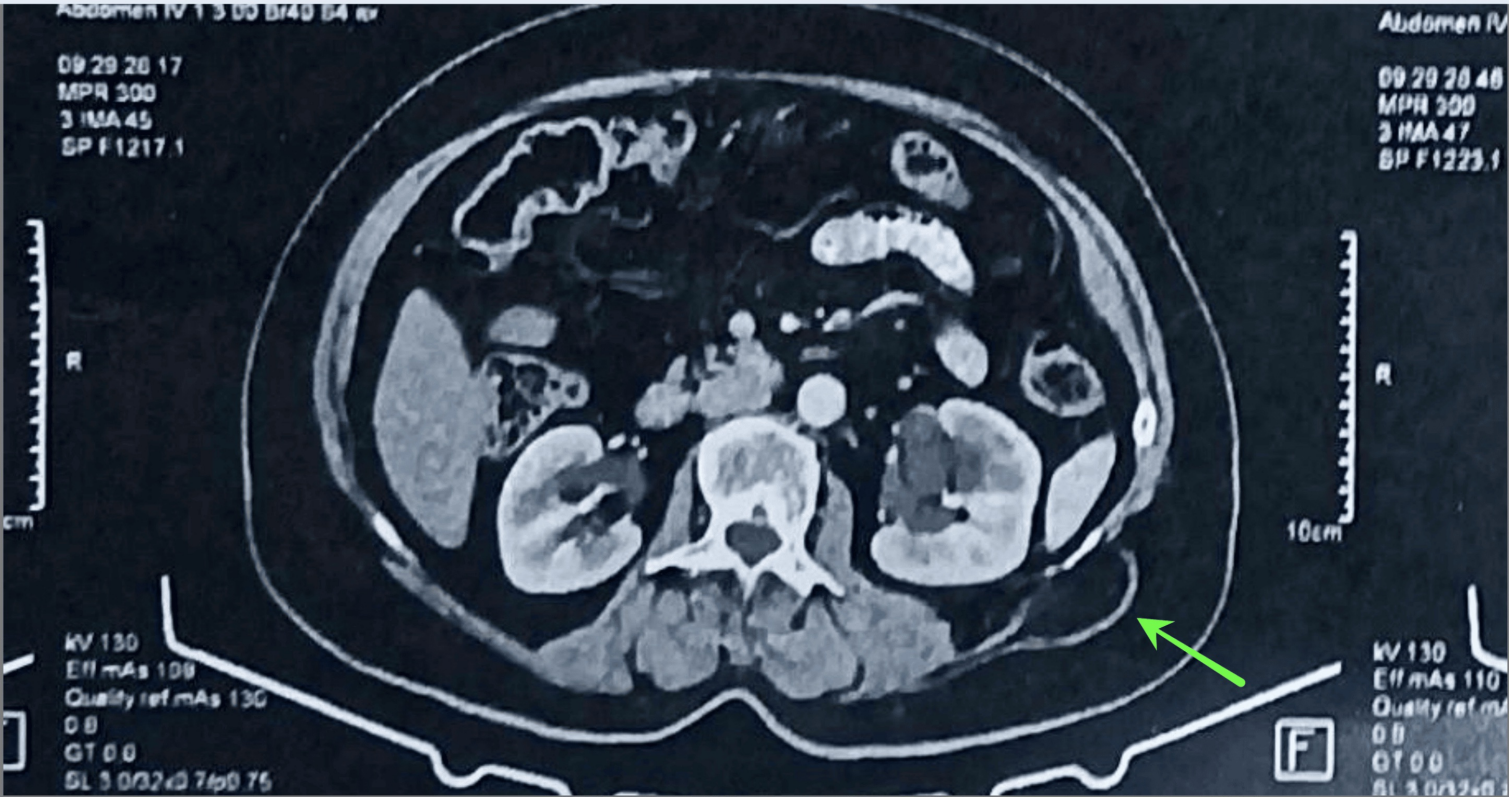
Computed tomography (CT) The CT scan showed a defect in the left lumbar region (green arrow) containing probably extraperitoneal fat. The hernia was suspected to be a Grynfeltt-Lesshaft hernia based on the CT scan and the location of the patient’s pain

A 56-year-old female patient presented to the outpatient clinic of our surgery department, complaining about chronic not continuous discomfort for about a year in a region between the left midaxillary line just below the costal margin and the left lumbar region. The patient had unremarkable medical history and when asked denied any history of trauma, had no previous surgeries, and did not mention chronic cough or constipation although she did have a physical job. Her BMI was calculated to be 26.3. Upon physical examination, no mass or defect could be observed or palpated, even when trying different positions in examining the patient or tests such as the Valsalva maneuver, or asking her to cough, both of whom were negative for local swelling or mass appearance. A CT scan was ordered to identify the cause of her symptoms (Figure 1).

**Figure 2:**
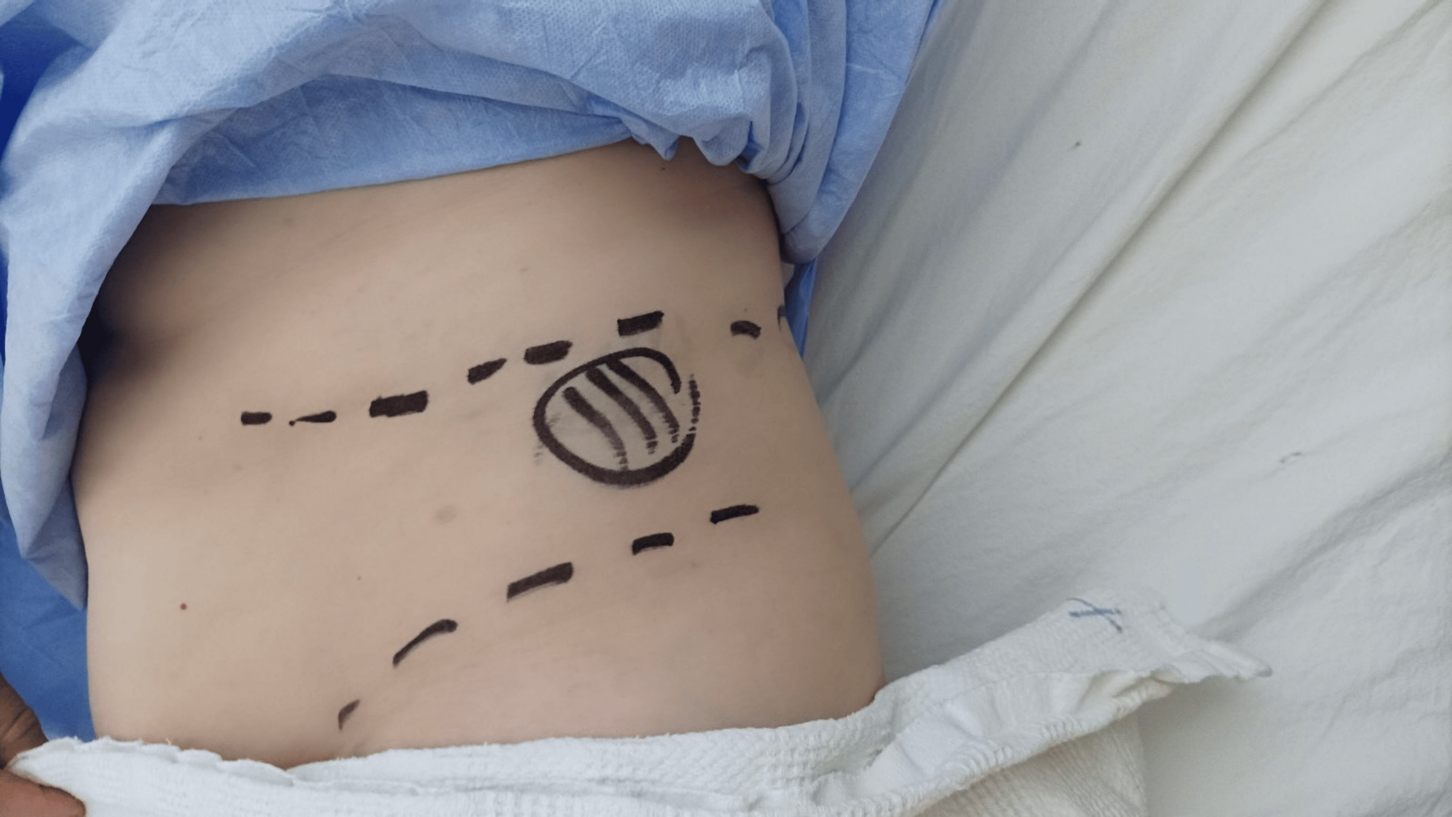
Preoperative marking of the site in which the patient felt pain. Also the costal line and the iliac crest margins were marked

The patient was then scheduled for surgical repair of her hernia (Figure 2). We opted for the laparoscopic approach since the defect couldn’t be palpated easily. The operation was performed under general anesthesia with the patient placed in the right lateral decubitus position. We used a 30^o^ laparoscope inserted from a 12 mm trocar placed approximately 4 cm left of the umbilicus, while two additional 5 mm trocars were inserted, one at the level of the left midclavicular line below the costal margin (left operating trocar) and one medially to the left iliac crest (right operating trocar), with a 10 cm distance from each other to allow triangulation of the operative instruments.

**Figure 3:**
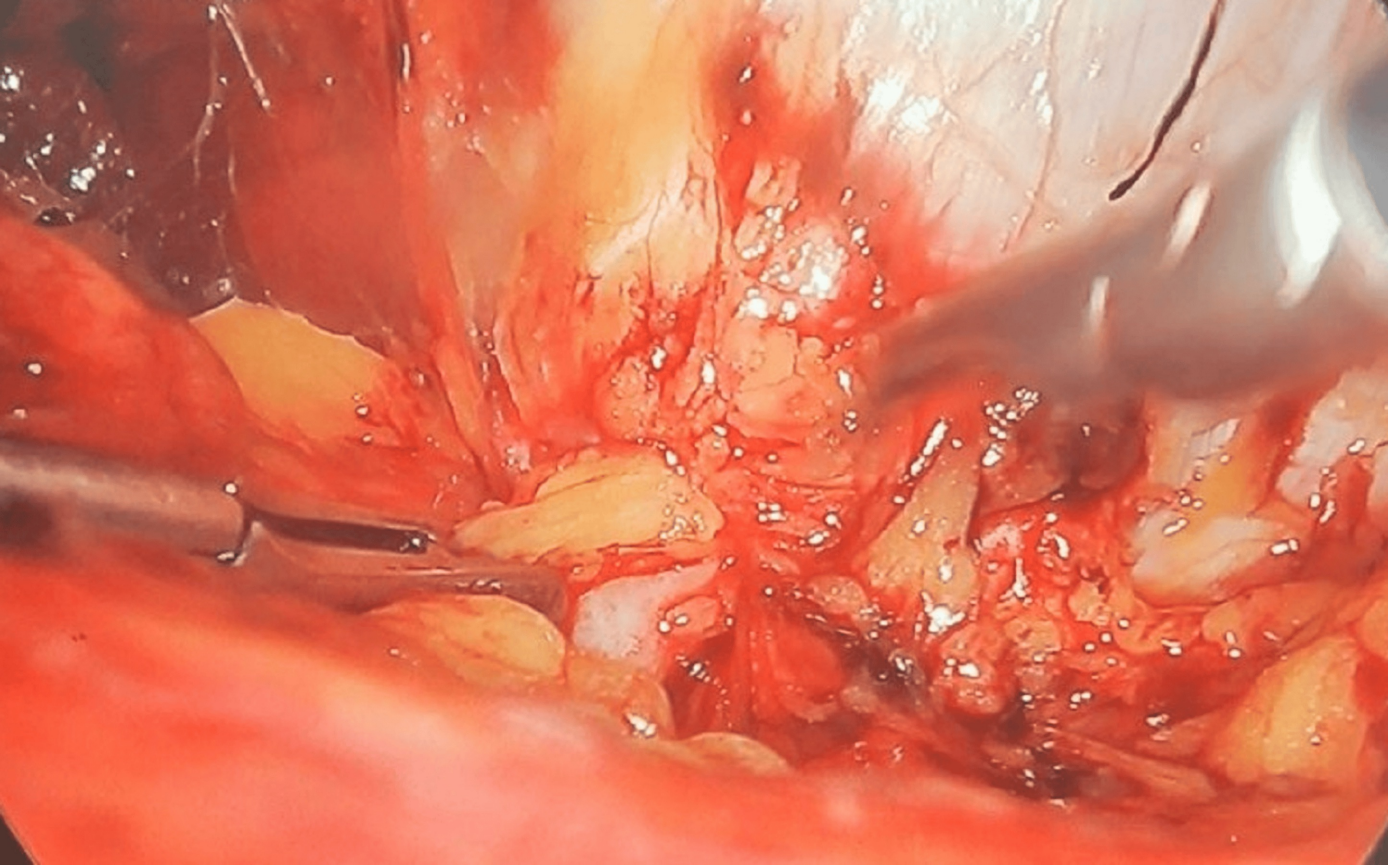
Adipose tissue protruding through the defect in the left lumbar region

**Figure 4:**
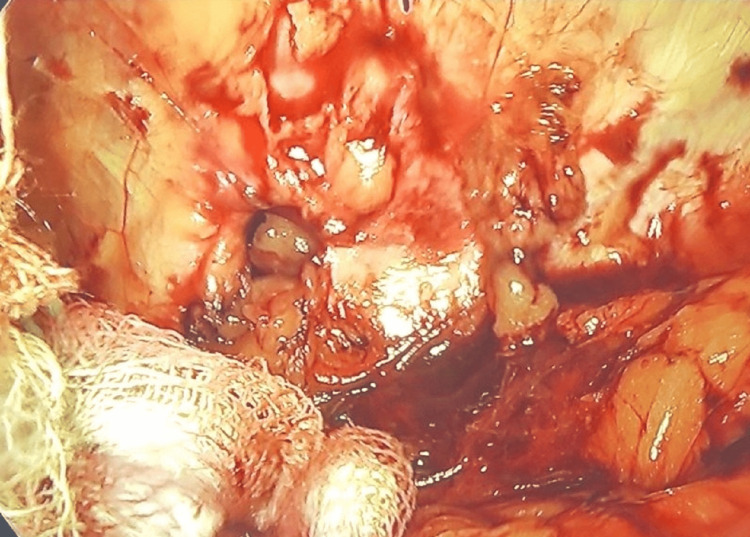
The abdominal wall defect after reducing hernia contents

The peritoneum of the left paracolic gutter was incised from the 11th rib superiorly to the iliac crest inferiorly. The peritoneum and retroperitoneal plane were dissected from the muscle wall in the lumbar region allowing the identification of a defect containing adipose tissue (Figure 3). We then reduced the contents of the hernia, allowing for a better visualization of the defect which was about 1.5 cm in diameter (Figure 4). 

**Figure 5:**
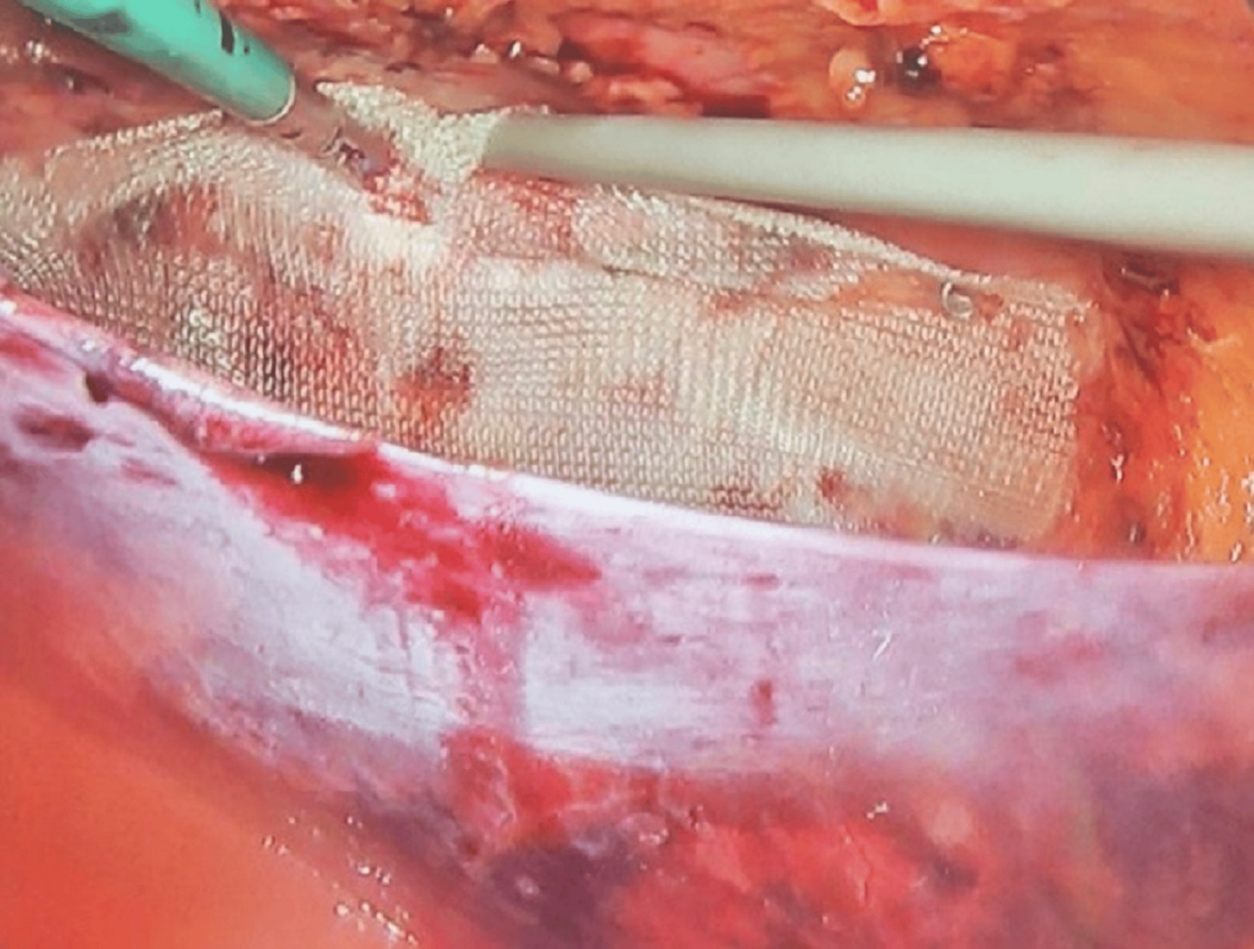
Mesh placement using staples between the muscle wall and the peritoneal flap

The gap was closed with running absorbable suture followed by a 10 x 10 cm polypropylene mesh placement, with a 4 cm overlap on each side, using staples (Figure 5). Special consideration was given to not injure any bone structures or surrounding nerves (ilioinguinal, iliohypogastric, etc.); thus, we avoided excessive staple placement. Subsequentially, closure of the peritoneum of the left paracolic gutter was achieved using staples. Similarly, aponeurosis of the 12 mm trocar was closed using 1-0 PDS sutures. 

The operation was concluded uneventfully, within one hour of anesthesia induction. There were no complications, and the patient was discharged on the first postoperative day. After 10 days, the patient reported only mild pain/discomfort around the surgical area that could be relieved with over-the-counter pain relievers. After 20 days in a second follow-up visit, she was completely asymptomatic.

## Discussion

Lumbar hernias, whether of the Grynfeltt-Lesshaft type or the Petit type, are rare, and they are not on top of the list in the differential diagnosis when it comes to back pain or a back mass. They can easily be mistaken for a more common diagnosis like a lipoma. Often, a Grynfeltt hernia is not evident as the large latissimus dorsi overlays the defect, making it hard to palpate [7]. Our patient falls in this category, and only the CT scan was able to confirm the diagnosis. The possibility of hernia strangulation is ever present which could lead to bowel obstruction or necrosis among other complications [8]. Possible herniated contents include extraperitoneal fat, the omentum, kidney, spleen, small intestine, or the colon. These defects will increase over time as in other hernia types which could worsen the symptoms, even if the hernia doesn’t become incarcerated. Upper lumbar hernias were described by Lesshaft (1870) and Grynfeltt (1866), and the first reported case was from Garangoet (1731) [9]. They are more common in men, aged 50-70 years, usually unilateral on the left side, and are caused by a rupture in the transversal fascia in correspondence to the Grynfeltt space [10-11]. About 20% of Grynfeltt hernias are congenital (due to embryogenetic defects such as anomalous vertebral and rib defects) and 80% are acquired; 55% of the acquired ones are either spontaneous or primary (atraumatic); and the remaining 25% are secondary, caused by injury or surgery [10-11]. While some cases have been reported in the literature, no large study has been implemented to establish the most prominent way of surgical management. Both the open and the laparoscopic techniques with the use of a mesh have a high rate of success, whereas techniques with closure under tension are being abandoned. In our case, we utilized both methods, as the size of the defect allowed a relatively comfortable primary closure and then secured the mesh in the preperitoneal space with sufficient overlapping of the defect. Generally, laparoscopic repairs of lumbar hernias were shown to be superior in length of hospital stay and postoperative complications but inferior in terms of intraoperative complications [12]. Other important advantages of laparoscopic repair include the better visualization of anatomical landmarks and the defects [13] and the possibility of a better mesh placement without the need for extensive fixation due to it being held in place by intraabdominal pressure [11] while achieving better aesthetic effects and lower surgical site infection rates [14]. In the laparoscopic preperitoneal approach, like our case, great care must be taken in order to avoid damaging the subcostal, iliohypogastric, and ilioinguinal nerves, especially in cases in which the mesh is placed between the peritoneum and the muscle wall because the defect should be dissected circumferentially from the peritoneum and extraperitoneal fat to allow mesh placement and fixation, also a possible time of nerve injury [15]. It is essential that surgeons with adequate laparoscopic experience are to perform such a procedure while utilizing all of the laparoscopic technique advantages.

## Conclusions

Grynfeltt hernia and Petit hernia are rare and require a careful evaluation by the physician for correct diagnosis and operative management. CT scan or MRI of the abdominal wall can be used for diagnosis and characterization in less evident cases such as our patient. As rare types of hernia, there is no gold standard surgical treatment for both open and laparoscopic repairs with various techniques being reported in the literature. Our case report demonstrates the need for an increased level of suspicion in patients with no obvious defect or with vague symptoms, and our impression is that laparoscopic surgical repair with preperitoneal mesh placement is a safe procedure with many advantages either intraoperatively or postoperatively. Patient selection and the experience of the surgical team are of utmost importance to achieve the best possible result and to prevent complications or recurrence. Our study has a limitation concerning this matter since we do not have a long-term follow-up on this patient yet. Further studies comparing open vs. laparoscopic approaches should be conducted for surgeons to decide the best type of repair for each case.
